# Inhibiting Effect of Inner Potential on Electroporation of Phospholipid Membranes Induced by Ionic Electrophoresis

**DOI:** 10.3390/ijms27031465

**Published:** 2026-02-01

**Authors:** Ping Ye, Haoyang Li, Kuiwen Zhao

**Affiliations:** School of Health Science and Engineering, University of Shanghai for Science and Technology, Shanghai 200093, China; iamyeping@usst.edu.cn (P.Y.); 233352386@st.usst.edu.cn (H.L.)

**Keywords:** molecular simulation, molecular dynamics, phospholipid membrane, electroporation, ion concentration, inner potential

## Abstract

Understanding electroporation at the molecular level is essential for advancing its biomedical applications, including drug delivery and tumor ablation. Among various influencing factors, the ionic environment, particularly ion concentration and type, plays a crucial role in modulating membrane behavior. In this study, we performed systematic molecular dynamics simulations to investigate how different ions affect the electroporation of phospholipid membranes. While moderate ion concentrations were found to accelerate pore formation by enhancing ion–membrane interactions, our results reveal that excessively high ion concentrations inhibit electroporation. Further analysis shows that this inhibition is primarily due to the formation of an inner potential, induced by the electrophoretic movement of ions under an applied field. This inner potential effectively weakens the transmembrane electric field, delaying or even preventing pore formation. Additionally, we demonstrate a strong correlation between ion charge concentration and electroporation time, regardless of ion species. These findings uncover a concentration-dependent shift in electroporation mechanisms and highlight the critical role of inner potential in regulating membrane permeability. This work provides valuable theoretical insights for the precise control and optimization of electroporation-based therapies.

## 1. Introduction

Electroporation is a technique that utilizes high-magnitude electric pulses to induce an increase in cell membrane permeability [1,2,3], and it has emerged as a widely used tool in biomedical fields such as gene transfection, cell fusion, and drug delivery [4,5,6]. The phenomenon of electroporation has a long history, with early observations dating back to 1754, when Nollet documented the effects of static electrical discharges on the skin [7]. The first observation of electroporation was reported in the 1950s on electrically stimulated membranes and was described as “membrane rupture” [8]. Subsequently, extensive studies demonstrated the applicability of electroporation in molecular biology and clinical medicine, particularly in the treatment of malignant tumors using irreversible electroporation (IRE) [9,10]. This technique has been shown to effectively ablate various types of cancer cells and tissues while preserving the extracellular matrix and critical structures [11,12].

In practical biological systems, both extracellular and intracellular fluids are rich in ions that regulate essential cellular functions, maintain electrolyte balance, and modulate membrane potential [13]. Accumulating experimental evidence indicates that the ionic environment plays a significant role in determining the efficiency of electroporation [14,15,16]. For example, cells treated with cationic molecules or dimethyl sulfoxide (DMSO) can be killed by IRE at moderate electric field strengths [17,18]. Grys et al. [19] reported that changes in phospholipid structure induced by cations increase cellular sensitivity to electric fields, demonstrating that locally applied cationic anesthetics can reduce the electric field strength required for IRE by approximately 50%, thereby markedly enhancing treatment efficacy.

From a mechanistic perspective, molecular dynamics (MD) simulations have become a powerful tool for investigating pore nucleation and membrane structural evolution under electric fields at the molecular level [20,21]. Previous MD studies have revealed key features of electroporation in single-component phospholipid bilayers, including water defect formation, pore expansion, and subsequent membrane rearrangement [22,23]. Mou et al. [24] further showed that sodium chloride exerts opposite effects at different stages of electroporation: ions promote pore initiation but destabilize pores after formation by interacting with polar lipid molecules at the pore edge, leading to accelerated pore closure. Similar ion–lipid interactions and membrane property modulations have also been reported for other chemical additives [25,26].

However, despite these advances, existing studies have mainly focused on how ions alter membrane structure or local lipid organization. For tumor ablation efficiency based on irreversible electroporation, a critical unresolved issue is how changes in the ionic environment of the solution modify the ion-induced inner potential and thereby regulate the effective electric field that drives pore formation. In particular, whether ion accumulation can generate an inner potential that counteracts the applied external field and suppresses pore formation remains poorly understood. This knowledge gap directly limits the accurate prediction of ablation efficiency in heterogeneous tumor microenvironments and hinders the rational optimization of clinical IRE treatment parameters.

To address this problem, we performed coarse-grained molecular dynamics simulations of phospholipid bilayer membranes in electrolyte solutions with varied ion concentrations and ion types. We measured and compared the electroporation time under different ionic conditions and analyzed the respective contributions of electrostatic interactions, ion migration, and the resulting inner potential to membrane destabilization. Finally, the relationship between ionic charge concentration and electroporation time was established across different cation species. These results provide new mechanistic insight into how the solution ionic environment regulates electroporation through electric field redistribution and offer a theoretical basis for the rational optimization of electroporation-based tumor ablation and other biomedical applications.

## 2. Results and Discussion

### 2.1. Equilibrium of Membrane Properties

**Figure 1:**
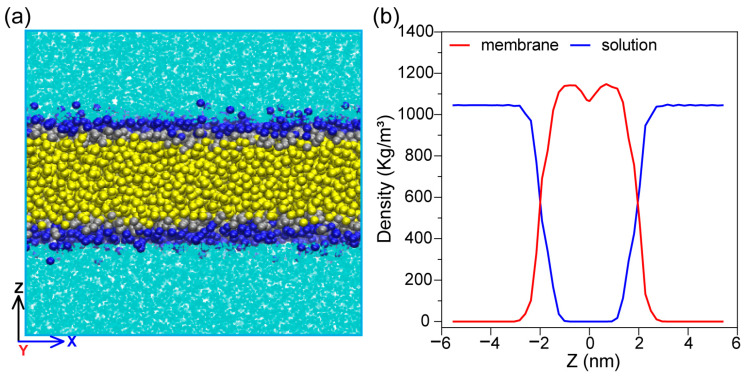
Information on pure water membrane system. (**a**) Equilibrated coarse-grained model of a DPPC phospholipid bilayer membrane in pure water. Dark blue and silver particles represent the DPPC phospholipid head, and yellow particles represent the DPPC phospholipid tails, and the light blue particles represent polar water molecules. (**b**) The density of each component in a pure water membrane system. The membrane density represents the density of the entire phospholipid molecule, including choline, phosphate, glycerol, and fatty acids.

The pure water membrane system is taken as an example below, and the molecular model is shown in Figure 1a. Dark blue, silver, and yellow particles represent the 1,2-dipalmitoyl-sn-glycero-3-phosphocholine (DPPC) phospholipid membrane, and the light blue particles represent polar water molecules. The dimensions of the entire system were 18.1 nm × 18.1 nm × 12.6 nm. The density of water was 1045 kg/m^3^ (Figure 1b), which is in line with previous studies [27].

**Figure 2:**
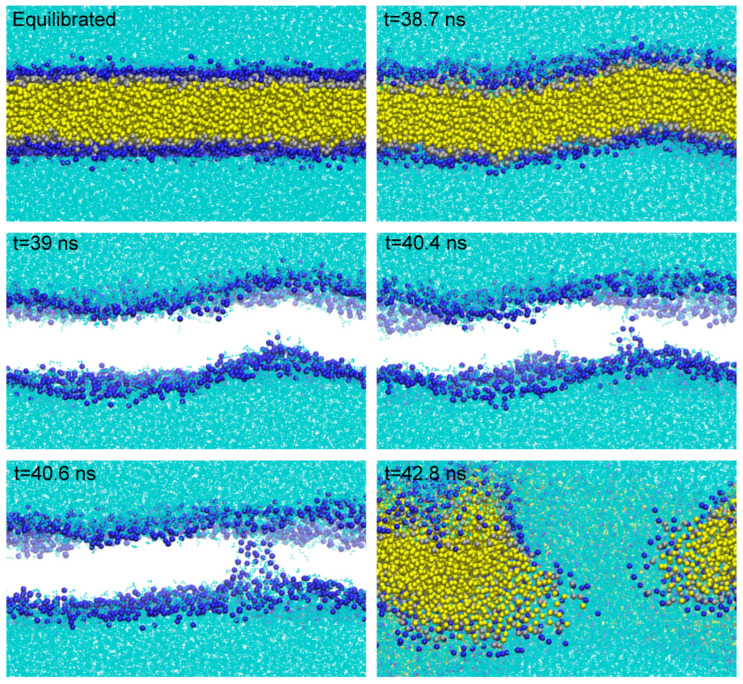
Pore formation process in the pure water membrane system under an external electric field. Snapshots of membrane electroporation under an applied electric field of 0.2 V/nm along the positive Z-direction. Equilibrated ~38.7 ns, the membrane shows significant deformation; 39 ns~40.4 ns, the water chain formed without clear connectivity; 40.6 ns~42.8 ns, water chain pore completely formed.

To better quantify the effect of ion concentration and type, we simulated a comparison system using pure water. The progress of pore formation is shown in Figure 2. First, the membrane shows significant deformation at 38.7 ns. From 39 ns to 40.6 ns, we hide the phospholipid tail (yellow particles) in order to more clearly observe the formation of water chains and the movement trend of the phospholipid head groups. At 40.6 ns, forming a distinct water chain, this time was recorded as the time when pore appeared [28].

### 2.2. Differences in Electroporation Time of Different Systems

**Figure 3:**
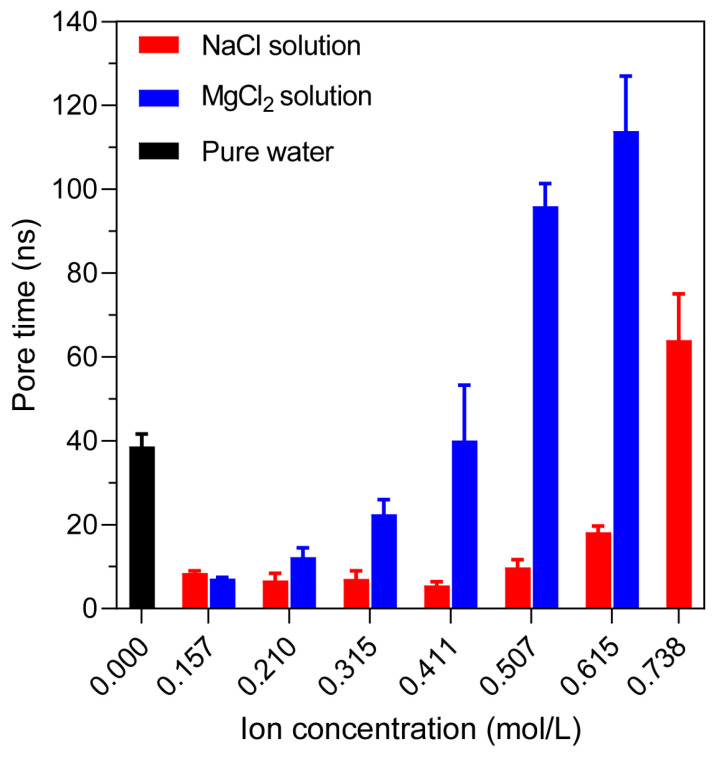
Pore formation time under different ion concentrations. Variation of electroporation time in systems containing different concentrations of NaCl and MgCl_2_ compared with the pure water system. The results are the average of five simulations.

Compared with the pure water system, the pore formation time was reduced from 40.4 ns to about 8.4 ns at 0.15 mol/L NaCl and 7.1 ns at 0.15 mol/L MgCl_2_. In NaCl solutions, pore formation occurred rapidly at low concentrations (0.157–0.411 mol/L), about 13–24% of the pure water value, but the times increased at higher concentrations, reaching 54.2–76.0 ns (1.4–1.9× pure water) at 0.738 mol/L. Similarly, the MgCl_2_ system exhibited a much stronger inhibitory effect even at moderate concentrations. At 0.738 mol/L, no pore formation was observed during the simulation time, indicating a complete inhibition of electroporation (Figure 3) [29]. These results demonstrate a strong ion-dependent modulation of membrane stability, particularly under the influence of divalent cations like Mg^2+^. Although individual realizations exhibit intrinsic variability, the relative dependence of electroporation time on ion concentration and ionic valence remains highly consistent.

**Figure 4:**
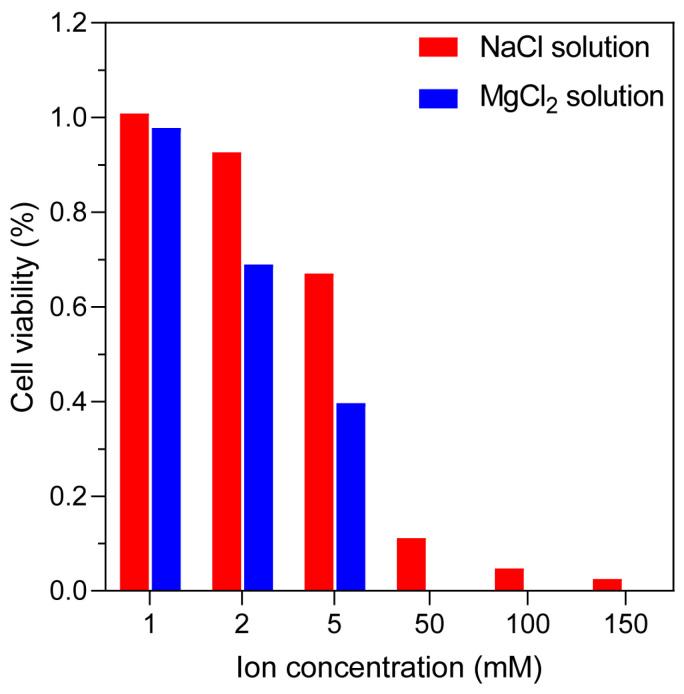
Cell survival rate under different ion concentrations. In vitro electroporation results of Hepa1-6 mouse hepatoma cells in NaCl and MgCl, solutions with varying concentrations. Cell survival decreases with increasing ionic concentration.

To provide biological support for the simulation results, we performed in vitro electroporation experiments using Hepa1-6 mouse hepatoma cells under different extracellular ionic conditions (NaCl and MgCl_2_ solutions) [30]. Detailed experimental procedures are provided in the Supplementary Material. Although the related parameters used in the experiments differ from those in the simulations, the results clearly demonstrate a consistent trend: as the ionic concentration in the solution increases, cell survival rate decreases (Figure 4). This trend aligns well with the outcomes predicted by the molecular dynamics simulations. It should be noted, however, that experimental validation at very high ion concentrations was not feasible due to the significant cytotoxic effects under such conditions, which would inherently compromise cell viability and confound the assessment of electroporation-specific outcomes. Therefore, the experiment is only to prove that the electroporation process is related to the ion valence state.

### 2.3. High Ion Concentration Inhibits Electroporation

We analyzed the extreme phenomenon of significantly prolonged electroporation time or even no pores appearing in the above experimental results from the perspective of ionic behavior. One crucial factor is the inner potential generated by the redistribution of ions across the membrane [31,32], which plays a critical role in determining the membrane’s electrical response and stability under an external field.

**Figure 5:**
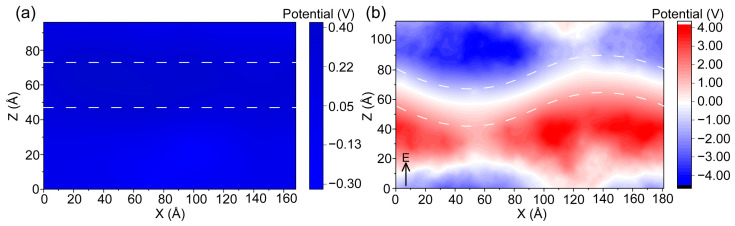
Ion-induced inner potential distribution. Inner potential distribution of ions in the simulation system. The dashed lines indicate the positions of the upper and lower leaflets of the phospholipid bilayer. (**a**) Inner potential distribution in the absence of an external electric field. (**b**) Inner potential distribution prior to pore formation under an applied electric field. E is the direction of the external electric field.

To better understand this effect, we analyzed the inner potential generated by ions before the appearance of the membrane pore. As shown in Figure 5a, in the absence of an applied electric field, the ions on both sides of the membrane exhibit relative symmetry, resulting in a nearly uniform inner potential across the system, as a comparison with the inner potential after application of an external electric field [33]. Although the charges on the membrane surface attract oppositely charged ions, forming localized charge layers on either side, this distribution is generally stable and does not lead to membrane disruption. However, upon the application of an external electric field, the induced directional migration of ions causes a non-uniform distribution of the inner potential, with increased asymmetry between the two sides of the membrane. When a constant uniform electric field is applied along the positive direction of the *Z*-axis, the inner potential map reveals distinct changes in the ion distribution across the membrane (Figure 5b). This redistribution of ions leads to the formation of a counter-electric field within the membrane region.

**Figure 6:**
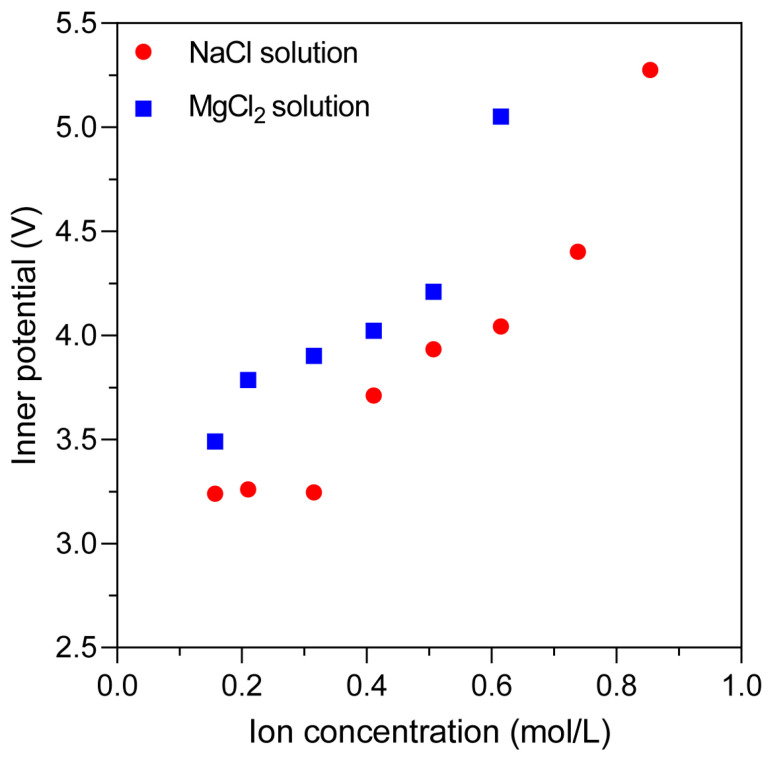
Inner potential under different ion concentrations. Dependence of ion-induced inner potential on ions concentration, showing that higher ion concentrations generate stronger inner potentials, which increasingly counteract the applied electric field and inhibit electroporation.

Electroporation requires overcoming a certain transmembrane energy threshold. Since the applied external electric field is constant, we are concerned that the presence of an ion-induced inner potential can partially offset the applied electric field, thereby weakening its effective strength at the membrane surface. To quantify this effect, we extracted the inner potential values under different ion concentrations (Figure 6) and found that as the ion concentration increases, the ion-induced inner potential becomes stronger, thereby providing greater resistance to the applied electric field. This enhanced shielding effect leads to a further increase in the energy barrier for electroporation, resulting in a prolongation of the time required for pore formation. This mechanistic insight explains the concentration- and ion-type–dependent suppression of electroporation, particularly in systems with high concentrations of divalent ions such as Mg^2+^.

**Figure 7:**
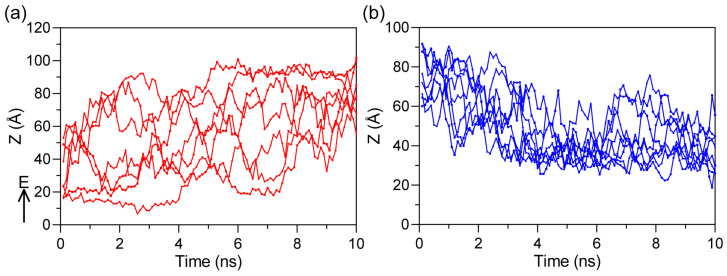
Ion migration trajectories before pore formation. The movement trend of ions under an applied electric field. (**a**) Na^+^ ions migrating along the field direction. (**b**) Cl^−^ ions migrating opposite to the field direction, illustrating electrophoretic ion redistribution.

Further research revealed that this distribution of inner potential is primarily caused by the migration of ions under the influence of the external electric field [34]. To investigate this phenomenon, we tracked the trajectories of all ions in the system before the formation of pore under periodic boundary conditions. The results indicate that upon application of an electric field along the positive direction of the *Z*-axis, cations and anions migrate directionally along and against the field direction, respectively (Figure 7). At this stage, since the phospholipid membrane remains intact at this time, ions cannot penetrate the membrane. Instead, ions accumulate on the membrane surfaces, forming charge layers. This directional migration behavior is a direct result of electrophoresis, where charged particles move under the force of the applied electric field [35]. As ions redistribute across the system, they generate a non-uniform inner potential, which modulates the local potential environment.

This inner potential plays a pivotal role in regulating electroporation: by partially offsetting the external electric field, it alters the net effective field and thereby inhibits pore formation. Consequently, ion concentration indirectly regulates electroporation time by modulating the strength and distribution of the inner potential through ion electrophoretic effects. The higher the ionic concentration, particularly in the presence of multivalent ions, the stronger the induced inner potential and the more pronounced the inhibition of membrane pore formation.

To elucidate the origin of the inner potential, we analyzed the ionic charge density distribution along the membrane normal. Before the application of the electric field, the charge density remained symmetric around the membrane. A notable finding was that cations (e.g., Na^+^) were distributed closer to the phospholipid headgroups than anions, indicating a preferential interaction [36].

**Figure 8:**
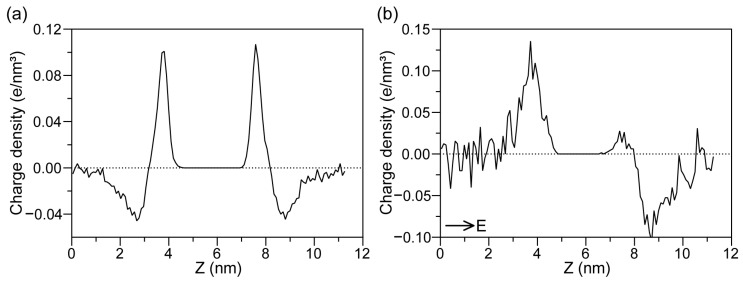
Ionic charge density distribution along the membrane normal. (**a**) Charge density profile before application of the electric field, showing a symmetric distribution around the membrane. (**b**) Charge density profile after applying the electric field but before pore formation, showing asymmetric accumulation of positive and negative charges on opposite sides of the membrane.

Upon the application of an electric field, this symmetry is disrupted: positive charges accumulate along the positive direction of the *Z*-axis, while negative charges shift in the negative direction, leading to a significant change in the ionic charge density profile of the system [37], as shown in Figure 8. This asymmetric charge accumulation directly contributes to the formation of the inner potential described above, further reinforcing the link between ion redistribution, inner potential, and the electroporation process. Although the applied electric field in this study exceeds experimental values, previous simulation studies have demonstrated that high fields primarily accelerate pore formation without qualitatively altering the underlying electroporation mechanism. Therefore, while the absolute pore formation times reported here should not be interpreted quantitatively, the relative trends with respect to ion concentration and ionic valence are expected to remain valid at lower field strengths.

### 2.4. Low Ion Concentration Promotes Electroporation

In contrast to the inhibitory effects observed at higher ionic concentrations, electroporation occurred significantly faster at lower NaCl concentrations (0.157–0.411 mol/L) [38]. This acceleration can be attributed to the interactions between the ions and the phospholipid membrane under the influence of an external electric field [39].

**Figure 9:**
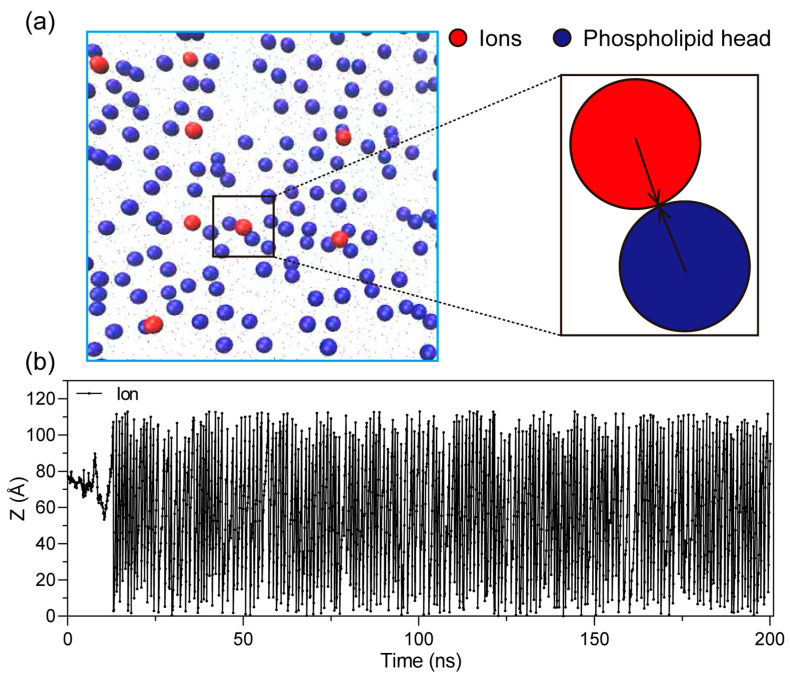
Schematic illustration of ion–membrane interactions at low ion concentrations. (**a**) Electrostatic interactions between migrating ions and charged phospholipid headgroups. Arrows indicate interactions. (**b**) Repeated ion collisions at the membrane surface during simulation, leading to local membrane perturbation and accelerated pore initiation.

When charged particles are present in the system, they exert electrostatic interactions with each other and with the charged components of the membrane [40]. Under the applied electric field, ions begin to migrate directionally and eventually collide with the membrane surface, as illustrated in Figure 9. Since the head groups of phospholipids carry charges, these collisions result in either electrostatic repulsion or attraction, depending on the polarity of the interacting ions.

Such interactions cause local disturbances in membrane structure, including changes in lipid packing and headgroup orientation. Over time, these perturbations accumulate under continuous electric field stimulation, weakening membrane integrity. This process facilitates the formation of transient defects that evolve into stable pores, thus accelerating the electroporation process at low ionic concentrations [41].

### 2.5. Effect of Charge Concentration

Given that both the inner potential and the electrostatic interactions discussed above are inherently linked to ionic charge, we further focused on the relationship between charge concentration and electroporation behavior [42].

The results showed that electroporation occurred more rapidly at lower MgCl_2_ concentrations (0.157–0.315 mol/L) than in the pure water system, consistent with the NaCl system. However, unlike NaCl, the delay in pore formation emerged earlier and more prominently in the MgCl_2_ system as the concentration increased. This can be attributed to the higher charge carried by Mg^2+^, which induces a stronger inner potential effect and more pronounced electrostatic interactions with the membrane [43]. Even at moderate concentrations, the presence of Mg^2+^ significantly attenuated the effective electric field, resulting in a lower electroporation efficiency compared to Na^+^.

**Figure 10:**
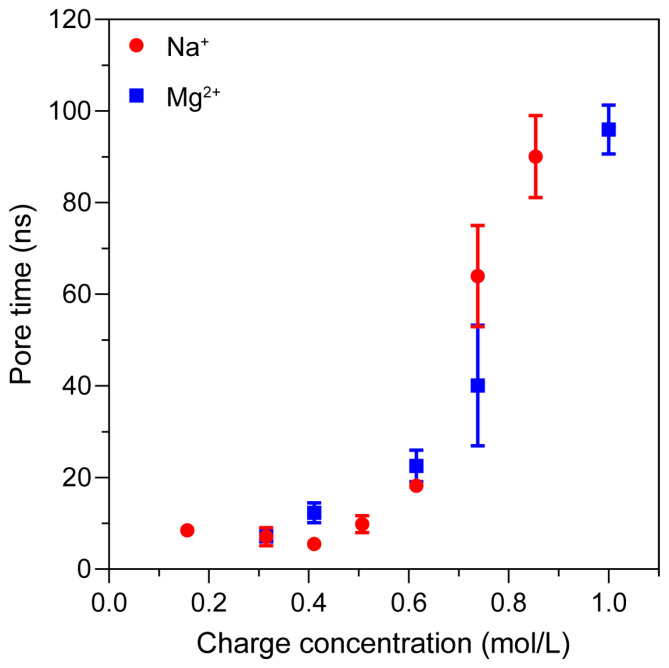
Relationship between charge concentration and pore formation time. The pore formation time correlates strongly with charge concentration rather than ion type.

To further examine the effect of charge concentration itself—independent of ion type—we analyzed the correlation between ionic charge concentration and pore formation time, as shown in Figure 10. Comparative analysis revealed that regardless of ion species, the overall trend in electroporation time correlates strongly with charge concentration. This suggests that the electroporation behavior is predominantly governed by the system’s charge concentration, and not merely by the presence of specific ions. In other words, as long as the charge concentration remains the same, similar electroporation trends can be observed.

**Figure 11:**
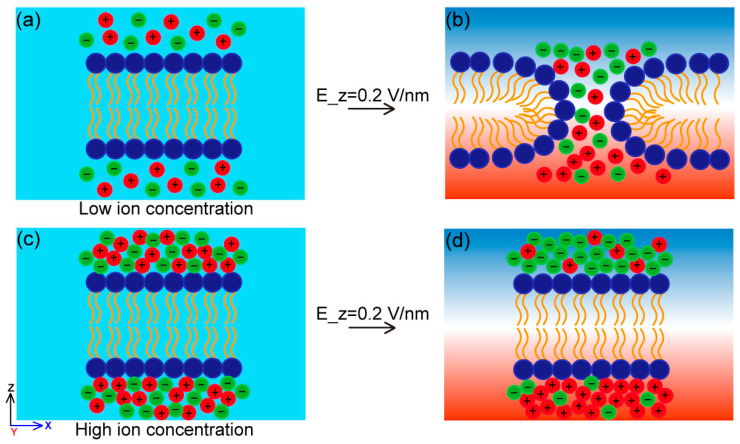
Schematic mechanism of ion-induced inner potential regulating electroporation. Phospholipid head groups and hydrophobic tails are shown in dark blue and yellow, respectively. Cations and anions are represented by red and green spheres. (**a**,**c**) show the system in the absence of an applied electric field, where ions are randomly distributed and the inner potential remains nearly uniform (light blue background). (**b**,**d**) show the system under an applied electric field along the positive Z-direction, where directional electrophoretic migration of ions leads to asymmetric charge accumulation near the membrane surfaces, resulting in a non-uniform inner potential distribution (blue and red background). At low ionic concentration (**a**,**b**), the external electric field is the primary driving force for pore formation. At high ionic concentration (**c**,**d**), ionic migrate generates an inner potential that offset the applied field and suppresses pore formation.

To facilitate intuitive understanding, a schematic illustration of the proposed mechanism is provided in Figure 11. In the absence of an external field, mobile ions are approximately uniformly distributed in the bulk electrolyte and generate only small local electrostatic fluctuations near the membrane (light blue background on the left). At low ionic concentration, ions weakly perturb the membrane and the applied external electric field dominates, leading to a large effective transmembrane field and rapid pore formation (Figure 11a,b). At high ionic concentration, directional ion migration under the external field causes asymmetric charge accumulation near the membrane surfaces, generating an ion-induced inner potential (blue and red background on the right) that partially counteracts the applied field. This screening effect reduces the effective electric field across the membrane and suppresses or delays pore formation (Figure 11c,d).

The present simulation results provide important implications for IRE in clinical tumor ablation. In practical treatments, tumor tissues are characterized by heterogeneous ionic compositions, elevated extracellular ion concentrations, and complex microenvironments caused by necrosis, inflammation, and vascular leakage [44,45]. Our findings suggest that such variations in ionic conditions may significantly alter the ion-induced inner potential, thereby reducing the effective electric field and delaying or suppressing pore formation. This mechanism offers a plausible physical explanation for the clinically observed variability in ablation outcomes, incomplete tumor coverage, and the requirement for higher pulse amplitudes in certain tumor types. In particular, tumors with high local ion concentrations or abnormal ionic distributions may exhibit increased resistance to electroporation due to enhanced inner potential shielding.

## 3. Materials and Methods

### 3.1. Molecular Dynamics Simulation Setup

In this study, 1024 DPPC molecules were selected and combined with a 4.3 nm-thick layer of water, and the model was constructed using the CHARMM-GUI modeling platform [46]. All systems were simulated using the GROMACS simulation package version 2023 at coarse-grained resolution using the Martini force field [37,47,48]. The leap-frog algorithm was utilized in this MD simulation to integrate the equations of motion. In the equilibrium process, the Parrinello–Rahman method was adapted to control the horizontal and vertical pressure on the membrane at 1 bar, with a dynamic step size of 20 fs and a total simulation time of 10 ns. The temperature was controlled at about 310 K by the Berendsen method, with a dynamic step size of 20 fs and a total simulation time of 100 ns. The spatial reference frame is such that the *x*- and *y*-axes are taken in the plane of the bilayer, whereas the *z*-axis is perpendicular to the membrane surface. This study used periodic boundary conditions (PBC) in three dimensions throughout the MD simulation process [49]. The entire simulation process uses the Verlet neighbor list generation method. The Van der Waals interaction was calculated by the Cut-off method, and was smoothly switched from 1.2 nm to 0, and electrostatic interactions were treated using the PME method in a distance of 1.4 nm [50]. All bonds involving hydrogen atoms were constrained using the LINCS algorithm. During the simulation, the length of the box in the X and Y directions is fixed to ensure that the area of the phospholipid part is maintained at 64 Å^2^ [51]. From this, the MD simulation stage is carried out, with a dynamic step size of 20 fs and a total simulation length of 200 ns. A static external electric field of 0.2 V/nm is applied in the positive direction of the *Z*-axis, which is non-physiological but necessary with the aim of observing significant membrane electroporation phenomena within the nanosecond-scale simulation time frame [23,52]. After the simulation, the Visual Molecular Dynamics (VMD) software version 1.9.3 was used to observe the condition of membrane electroporation [53] to observe ion motion by writing TCL scripts, and the GROMACS analysis tool was used to analyze the trajectory. Pore formation occurs when water molecules on both sides of the membrane meet to create water chains that traverse the bilayer.

To understand the effect of the solution environment on the membrane electroporation mechanism, we simulated the pure water membrane, the membrane containing NaCl, and the membrane containing MgCl_2_, and adjusted the concentration. For systems containing NaCl or MgCl_2_, we used the genion command to randomly replace the water in the solvent part of the pure water phospholipid membrane system and ensure that the system is electrically neutral. To verify the contribution of ions with different valences to membrane electroporation under an electric field, we simulated the effect of MgCl_2_, which contains divalent cations (Mg^2+^), and compared it with NaCl under the same molar concentration. Ion concentrations were adjusted to 0.157–0.854 mol/L for NaCl and 0.157–0.615 mol/L for MgCl_2_. Each system was simulated five times under the same initial conditions to account for stochastic variability. All simulation parameters, including force field settings, electrostatic treatments, and thermostats, were consistent across experiments to ensure comparability.

### 3.2. Inner Potential Calculation

The ion-induced inner potential was calculated from the spatial distribution of mobile ions using Poisson’s equation [54]. Only solution ions (Na^+^, Cl^−^, and Mg^2+^) were included. For each trajectory frame, ionic charges were mapped onto a three-dimensional grid with a spacing of 1 Å, and the charge density was averaged over time [55]. The electrostatic potential (*r*) was then obtained by solving

(1) $$\nabla^{2}\emptyset \left(r\right)=-\frac{\rho \left(r\right)}{\varepsilon_{0}}$$

where ρ(r) is the time-averaged ionic charge density and $\varepsilon_{0}$ is the vacuum permittivity, using a fast Fourier transform (FFT) method under periodic boundary conditions in all directions. We set the potential at infinity (or away from the bulk region of the membrane) to zero. To characterize the potential distribution relative to the membrane, we averaged along the y direction to obtain two-dimensional potential maps. The inner potential is defined here as the ion-induced electrostatic potential contribution obtained from this averaged profile, excluding the externally applied uniform electric field. This quantity reflects the internal electric field generated by asymmetric ionic accumulation during electrophoretic migration [56]. The ionic charge density distributions were derived from the GROMACS analysis tool.

### 3.3. In Vitro Electroporation Experiments

To complement the molecular dynamics simulations, in vitro electroporation experiments were performed using mouse hepatoma Hepa1-6 cells to evaluate the influence of ionic concentration and ion valence on cell survival.

Electroporation was performed using a nanosecond pulsed electric field generator with a field strength of 1 kV, pulse width of 500 ns, repetition frequency of 400 Hz, and 2000 pulses. Voltage and current waveforms were monitored using an oscilloscope. Cells were harvested at approximately 85% confluency and resuspended in NaCl solutions (1, 2, 5, 50, 100, and 150 mM) or MgCl_2_ solutions (1, 2, and 5 mM). Aliquots of 400 µL were transferred into electroporation cuvettes. After treatment, cells were mixed with an equal volume of complete medium, seeded into 96-well plates, and incubated for 24 h. Cell viability was quantified using a CCK-8 assay by measuring absorbance at 450 nm [57].

## 4. Conclusions

In this study, we used MD simulations to investigate the effects of different ion types and concentrations on the electroporation of phospholipid bilayer membranes. Our results show that electroporation is significantly inhibited at high ionic concentrations, mainly due to the formation of an ion-induced inner potential that counteracts the applied external electric field.

Under an external electric field, ions undergo directional electrophoretic migration, leading to asymmetric charge accumulation near the membrane surfaces. This process generates a non-uniform inner potential that reduces the effective electric field acting on the membrane. This inhibitory effect is particularly strong in the presence of divalent ions (e.g., Mg^2+^), which produce a much stronger inner potential even at moderate concentrations and may completely prevent pore formation within the simulation time. In contrast, at low ionic concentrations, ions facilitate electroporation by perturbing the local structure of phospholipid headgroups through electrostatic interactions, thereby accelerating membrane destabilization. Comparative analyses further indicate that electroporation time correlates more strongly with ionic charge concentration, highlighting the dominant role of charge-induced inner potential.

These findings establish inner potential as a key physical factor governing membrane electroporation and provide a mechanistic explanation for the variable efficiency of irreversible electroporation observed in different ionic environments, such as tumor tissues. Our results suggest that controlling ionic composition and charge concentration may represent an effective strategy to optimize electroporation efficiency for clinical applications including tumor ablation and drug delivery.

## Data Availability

Data for this article, including input files, simulation parameters, are available at the Science Data Bank at https://doi.org/10.57760/sciencedb.27941.

## Supplementary Materials

The supporting information can be downloaded at: https://www.mdpi.com/article/10.3390/ijms27031465/s1.
