# Supplementary material for: Inhibiting Effect of Inner Potential on Electroporation of Phospholipid Membranes Induced by Ionic Electrophoresis
Source: Int J Mol Sci. 2026 Feb 1;27(3):1465. doi: 10.3390/ijms27031465 (PMC12898162; doi:10.3390/ijms27031465)
Supplement: Supplementary file 1 [file ijms-27-01465-s001.zip › ijms-4081686-supplementary.pdf]

Supplementary Materials

To complement the molecular dynamics (MD) simulations of phospholipid membrane electroporation under varying solution conductivities, we conducted in vitro electroporation experiments using mouse hepatoma Hepa1-6 cells. The experimental results provide a biological correlate to the computational findings.

Cell Culture and Electroporation:

The mouse hepatoma cell line Hepa1/6 was obtained from the Chinese Academy of Sciences (Shanghai, China), this cell line is widely utilized in oncology research and possesses characteristics suitable for investigating electroporation effects. Cells were routinely cultured in Dulbecco's Modified Eagle Medium (DMEM; Gibco, USA) supplemented with 10% heat-inactivated fetal bovine serum (FBS; Capricorn) and 1% penicillin/streptomycin (Gibco, USA). Three days prior to the experiments, Hepa1-6 cells were seeded and maintained in a humidified incubator at 37°C with 5% CO_2 until they reached a confluency exceeding 85% and exhibited robust morphological status.

For the electroporation experiments, a high-frequency nanosecond pulse generator was employed, capable of delivering pulses with voltages up to ±2 kV, pulse widths ranging from 100 ns to 10 µs, and repetition frequencies from 1 to 400 Hz. The number of applied pulses could be varied as required. Connect oscilloscopes, voltage and current probes, and fixed clamp slots, etc. In this experiment, the pulsed electric field strength unit is set to 1 KV. The other parameters are fixed at frequency 400Hz, pulse width 500ns, pulse number 2000 times, and finally the pulsed electric field is applied. Use an oscilloscope to detect pulse emission and measure waveforms, currents, and voltages[1].

In our experiments, the solutions used consisted of NaCl (at concentrations of 1, 2, 5, 50, 100, and 150 mM) and $MgCl_2$ (at 1, 2, and 5 mM). First, cells were collected and resuspended in the respective solutions to obtain a uniform suspension at a density of 5×10^5 cells/mL, and then a volume of 400 µL of the cell suspension was transferred into an electroporation cuvette. After applying the electroporation stimulus, the treated cells were immediately resuspended in an equal volume of DMEM complete medium. Subsequently, the resuspended cells were evenly distributed into a 96-well cell culture plate at 100 µL per well and cultured uniformly in a 37°C incubator for 24 hours. Following this, 10 µL of CCK-8(Beyotime) reagent was added to each well, thoroughly mixed, and the plate was incubated again at 37°C for 4 hours. The optical density (OD) of each well was then measured at a wavelength of 450 nm

using a microplate reader to quantify the growth and proliferation status of the Hepa1-6 cells.

Although the solution concentrations used in the experiments differ from those in the simulations, the results clearly demonstrate a consistent trend: as the ionic concentration in the solution increases, cell survival rate decreases (Figure 4). This trend aligns well with the outcomes predicted by the molecular dynamics simulations. It should be noted, however, that experimental validation at very high ion concentrations was not feasible due to the significant cytotoxic effects under such conditions, which would inherently compromise cell viability and confound the assessment of electroporation-specific outcomes.

1. Ye, P.; Li, H.Y.; Zhang, M.Y. Irreversible Electroporation of AC16 Cardiomyocytes Induced by Nanosecond High Voltage Pulse In Vitro. *J. Cardiovasc. Electrophysiology.* **2025**, 37, 49-59. https://doi.org/10.1111/jce.70163.
